# Aberrant iPSC-derived human astrocytes in Alzheimer's disease

**DOI:** 10.1038/cddis.2017.89

**Published:** 2017-03-23

**Authors:** V C Jones, R Atkinson-Dell, A Verkhratsky, L Mohamet

**Affiliations:** 1 The University of Central Lancashire, Preston PR1 2HE, UK; 2 The University of Manchester, Manchester M13 9PT, UK; 3 Achucarro Center for Neuroscience, IKERBASQUE, Basque Foundation for Science, Bilbao 48011, Spain

## Abstract

The pathological potential of human astroglia in Alzheimer's disease (AD) was analysed *in vitro* using induced pluripotent stem cell (iPSC) technology. Here, we report development of a human iPSC-derived astrocyte model created from healthy individuals and patients with either early-onset familial AD (FAD) or the late-onset sporadic form of AD (SAD). Our chemically defined and highly efficient model provides >95% homogeneous populations of human astrocytes within 30 days of differentiation from cortical neural progenitor cells (NPCs). All astrocytes expressed functional markers including glial fibrillary acidic protein (GFAP), excitatory amino acid transporter-1 (EAAT1), S100B and glutamine synthetase (GS) comparable to that of adult astrocytes *in vivo.* However, induced astrocytes derived from both SAD and FAD patients exhibit a pronounced pathological phenotype, with a significantly less complex morphological appearance, overall atrophic profiles and abnormal localisation of key functional astroglial markers. Furthermore, NPCs derived from identical patients did not show any differences, therefore, validating that remodelled astroglia are not as a result of defective neural intermediates. This work not only presents a novel model to study the mechanisms of human astrocytes *in vitro*, but also provides an ideal platform for further interrogation of early astroglial cell autonomous events in AD and the possibility of identification of novel therapeutic targets for the treatment of AD.

All diseases, including neurological disorders, can be broadly defined as homeostatic failures within tissues, organs or systems. Astroglia are highly heterogeneous neural cells primarily responsible for homeostasis and neuroprotection in the CNS. Astrocytes are fundamental for synaptogenesis and synaptic maintenance; they control ion homeostasis in the CNS interstitium and are indispensable for turnover of major neurotransmitters, such as glutamate, GABA and adenosine.^1, 2^ In addition, astroglial cells are endowed with an evolutionary conserved defensive programme known as reactive gliosis, which develops in response to CNS lesions and is manifested by a spectrum of disease-specific cellular responses including hypertrophy and upregulation of intermediate filaments.^3, 4^ Astrocytopathy is a central element of neurological disorders and, depending on the disease context, astrocytes undergo complex changes, which vary from astroglial atrophy with loss of function, to pathological remodelling or reactivity and may develop alone or in combination.^5, 6, 7, 8^ In Alzheimer's disease (AD) animal models, atrophic astrocytes have been detected at the earliest stages of the disease, with hypertrophic reactive astrocytes appearing later in disease progression, in response to their proximity to extracellular accumulations of *β*-amyloid (A*β*).^9, 10, 11, 12^ Similarly, signs of astroglial reactivity and atrophy were detected in AD patients by positron emission tomography using ^11^C-deterium-l-deprenyl.^13^ These observations led to a hypothesis of glial paralysis being a fundamental factor in evolution of AD.^14^

The sporadic (late-onset) form of AD (SAD), which is without significant non-Mendelian genetic bias, dominates human pathology. Nevertheless, most of our knowledge of AD derives from studies that utilise cell- and animal-based models of the clinically rare, early-onset, dominantly inherited familial AD (FAD). The cellular pathology of astroglia in the context of human AD remains enigmatic, mainly because of severe limitations of animal models, which, although reproducing some pathological features of the disease, do not mimic its progression in full. Neuronal loss and cognitive deficits, which are the hallmarks of AD in humans, are limited in animal models^15^ and there is an increasing body of evidence showing that significant differences exist between rodent and human astrocytes.^16^ Although induced pluripotent stem cell (iPSC) technology can be used to investigate human astrocyte development and function, only a limited number of studies have described homogeneous generation of healthy astrocytes.^17, 18, 19, 20, 21, 22^ Previous work has shown that neurones differentiated from SAD- and FAD-iPSCs resemble pathologically affected cells *in vivo* and express key disease hallmarks;^23, 24, 25^ however, there is still a pressing need for tools to provide robust, homogeneous astrocyte populations to reveal astroglial contribution to neurodegenerative diseases. Here we report the generation of enriched mature astroglia from human iPSCs derived from patients with confirmed FAD and SAD as well as from a healthy control. We found that, although astrocytes derived from AD patients express the same canonical markers of mature healthy astrocytes, their morphological appearance and cellular phenotype is significantly distorted. Notably, we did not observe any differences in early neuronal commitment in early AD-derived neurones compared with healthy controls, indicating the cell autonomous pathological potential of astroglia and further confirming that cellular pathology does not stem from defective neural progenitors.

## Results

### Generation of neural cell lineages

**Figure 1:**
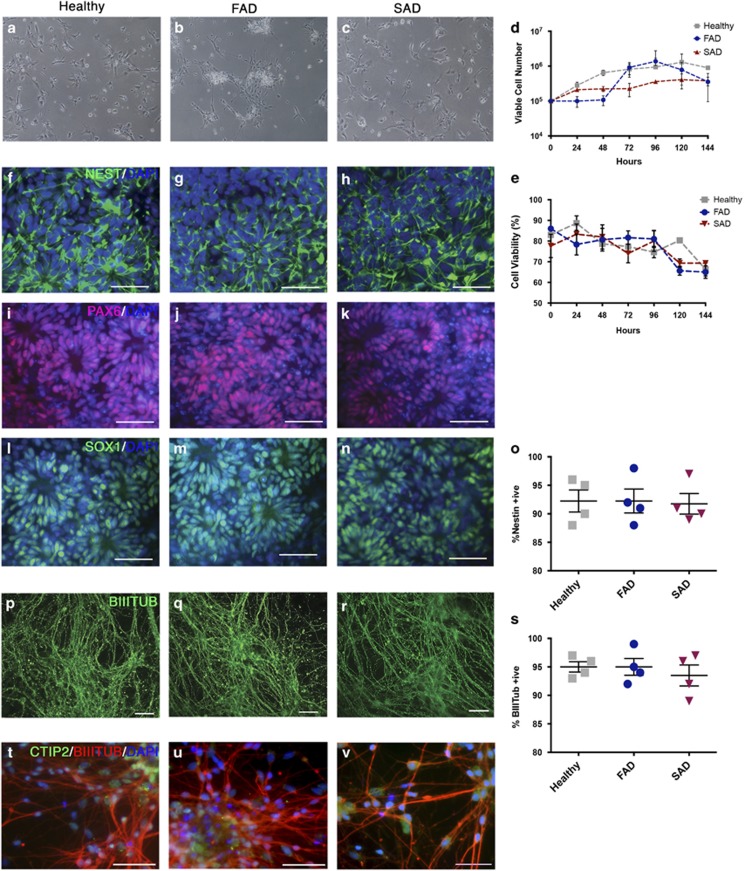
Directed differentiation of healthy and AD-neural progenitor cells into cortical neurones. (**a**–**c**) NPCs were seeded at 1 × 10^5^ per well and propagated in monolayer culture for 6/7 days. FAD and SAD cortical NPCs showed indistinguishable culture morphology with healthy (control) NPCs (*N* = 5 per cell line). (**d** and **e**) No significant differences in NPC growth rates were identified (*N*=4 per cell line, two-way Kruskal–Wallis *P*=NS). (**f**–**n**) IPS-derived NPCs from healthy (control), FAD and SAD patients were assessed for canonical marker expression. Progenitor cells formed polarised rosettes expressing nestin (green; **f**–**h**), PAX6 (red; **I**–**k**) and SOX1 (green, **l**–**n**). (**o**) No significant difference in nestin+ cells was observed between healthy and AD cell lines (*N*=4 per cell line, ANOVA, F_(2,9)_=0.022, *P*=NS). (**p**–**r**) Under terminal neuronal differentiation conditions for 35–40 days, all patient samples showed positive expression of the neural marker *β*-III-tubulin (green). (**s**) No significant difference in the proportion of *β*-III-tubulin+ neurones between any individual (*N*=4 per group, ANOVA, F_(2,9)_=0.128, *P*=NS). (**t**–**v**) Expression of the mature deep-layer cortical neuronal marker, CTIP2 was observed throughout cultures from each patient (green). Scale bars, 50 *μ*m

Cortical NPCs were propagated in monolayer culture and maintained as described in ref. 24. Both FAD and SAD NPCs showed indistinguishable culture morphology to healthy NPCs (*N*=5 per cell line; Figures 1a–c). Although a degree of variability in cell proliferation and viability was observed between cells from each individual, no significant differences in NPC growth rates were identified (*N*=4 per cell line, two-way Kruskal–Wallis, Figures 1d and e). Phenotypic identification of NPCs showed positive expression of the transcription factors SOX1 and PAX6, and the filamentous marker, nestin (Figures 1f–n). However, no significant difference in NPC marker expression was observed irrespective of genotype (quantified by the proportion of immunoreactive nestin+ cells; *N*=4 per cell line, analysis of variance (ANOVA), F_(2,9)_=0.022, *P*=NS, Figure 1o). Furthermore, under terminal neuronal differentiation conditions for 35–40 days, all patient samples showed positive expression of the neural marker *β*-III-tubulin (Figures 1p–r). No significant difference was detected in the efficiency of early neuronal induction (as measured by the proportion of *β*-III-tubulin+ neurones) between any individual (*N*=4 per cell line, ANOVA, F_(2,9)_=0.128, *P*=NS, Figure 1s). Expression of the mature deep-layer cortical neuronal marker, CTIP2 was observed throughout the cultures from each patient, indicating maintenance of cortical identity following 40 days in culture (Figures 1t–v). In a separate study, we also show that cortical neurones derived from identical FAD and SAD patients exhibit elevated pathological A*β*-42 secretion and hyperphosphorylated tau species compared with healthy individuals, therefore validating an AD phenotype in these cells (data not shown). The results presented herein reveal that both SAD and FAD NPCs retain their characteristic morphology, expression of canonical markers and are able to generate mature cortical neurones with the same efficiency as healthy derived NPCs, Importantly, therefore, any changes in astroglia are not as a result of defective neuronal intermediates.

**Figure 2:**
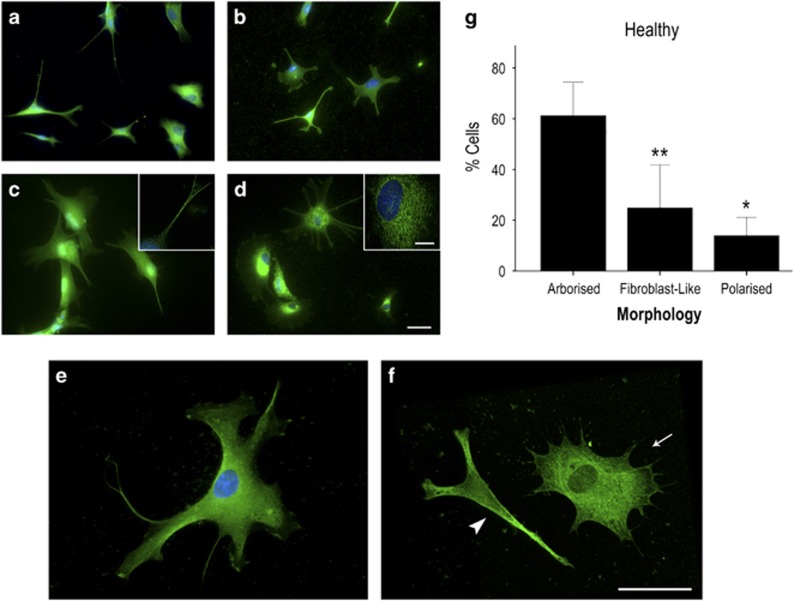
Induced astrocytes derived from healthy patient fibroblasts express mature astrocyte markers and exhibit varied morphologies. Induced astrocytes were generated in 30 days and confirmed by positive immunoreactivity to the functional markers: GFAP (**a**), S100B (**b**), EAAT1 (**c**) and GS (**d**). Immunostaining is shown in green, DAPI counterstained nuclei are shown in blue. Heterogeneity of morphology within the population of induced astrocytes was evident, with cells falling into three broad categories when stained for GFAP: arborised cells (**e**); polarised cells (**f**, arrowhead); or fibroblast-like, process-devoid cells (**f**, arrow); summarised in (**g**). **P<0.005, *P<0.05. Scale bars, 50 *μ*m (inset, 20 *μ*m)

To independently evaluate astroglial commitment, we adapted a defined differentiation protocol^18^ to generate purified astroglial cultures. We included additional culture supplementation of EGF and insulin, which have been shown to increase glial commitment, primary astrocyte proliferation and sub-culturing *in vitro*.^26, 27^ Astrocytes were generated within 30 days following induction from cortical NPCs in each cell line as confirmed by positive immunoreactivity to the canonical marker, GFAP and mature astrocyte markers; EAAT1, S100B and GS (Figures 2a–d). Quantification of GFAP, EAAT1, S100B and GS immunoreactive cells revealed near-pure expression in all astrocytes and no significant differences were observed between astrocytes derived from healthy control, FAD and SAD samples (*N*=5 differentiations per cell line (minimum 120 cells per group), ANOVA, Supplementary Figures S1A–D). We also examined astrocyte proliferation and viability (day 14 following induction) in both AD and control cells. Despite some variability in growth rates from each individual, no significant differences were observed in overall astrocyte proliferation or viability (*N*=3 per cell line, two-way Kruskal–Wallis, Supplementary Figures S1E and F). These results demonstrate that both *PSEN1* mutant and ApoE4^+/+^ NPCs are capable of generating astroglia *in vitro* and retain expression of markers consistent with mature astrocytes *in vivo*.

### Altered cellular heterogeneity in FAD and SAD astrocytes

**Figure 3:**
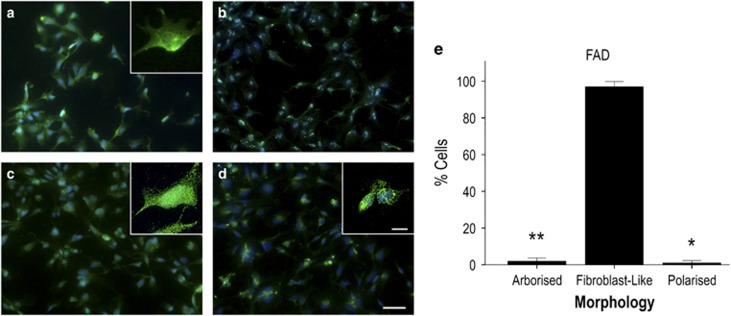
Induced astrocytes derived from *PSEN1* M146L FAD patient fibroblasts express normal astrocyte markers but show reduced morphological heterogeneity compared with healthy cells. Induced astrocytes were immunostained for GFAP (**a**), S100B (**b**), EAAT1 (**c**) and GS (**d**). Immunostaining is shown in green, DAPI counterstained nuclei are shown in blue; >95% of observed cells were positive for all markers tested. The significant majority of FAD astrocytes display a fibroblast-like, process-devoid appearance (summarised in **e**). **P<0.005, *P<0.05. Scale bars, 50 *μ*m (inset, 20 *μ*m)

**Figure 4:**
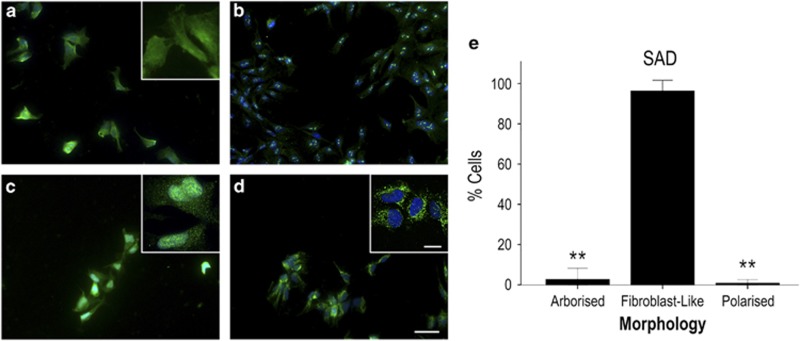
Induced astrocytes derived from *ApoE4*^+/+^ SAD patient fibroblasts express classical astrocyte markers but show reduced morphological heterogeneity compared with healthy cells. Induced astrocytes were immunostained for GFAP (**a**), S100B (**b**), EAAT1 (**c**) and GS (**d**). Immunostaining is shown in green, DAPI counterstained nuclei are shown in blue. All observed cells were positive for all markers. A significant majority of SAD astrocytes displayed a fibroblast-like appearance, indicating limited heterogeneity of morphology compared with healthy controls; summarised in (**e**). **P<0.005. Scale bars, 50 *μ*m (inset, 20 *μ*m)

**Figure 5:**
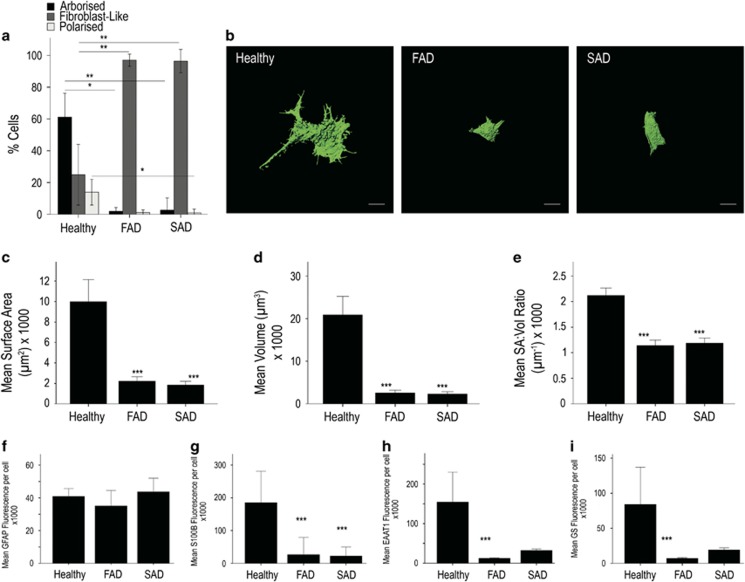
Astrocytes derived from *PSEN1* M146L FAD and *ApoE4*^+/+^ SAD patients exhibit significant atrophy when compared with those from healthy patients as revealed by visual binning according to overall morphology (**a**). Exemplar 3D IsoSurface renders constructed from serial confocal z-stacks display clear differences in cell size and overall morphology (**b**). Scale bar, 10 *μ*m. Quantification of cells using these renders by way of surface area (**c**), cell volume (**d**) and SA:Vol ratio (**e**) reveal significant differences in all aspects of cellular morphology between healthy and diseased astrocytes. Quantification of mean fluorescence intensity per immunoreactive cell reveals no significant difference in GFAP staining intensities between AD and control astrocytes (**f**) but S100B, EAAT1 and GS intensities are reduced in both FAD and SAD cells (**g**–**i**, respectively). ***P<0.001, **P<0.005, *P<0.05

Our initial observations revealed considerable differences in astrocyte morphology in cells derived from AD-NPCs compared with healthy controls. In healthy astrocytes, GFAP staining intensities did not differ from cell to cell, but overall morphology of individual cells across the population varied significantly (Kruskal–Wallis; H_(2)_=13.161, *P*=0.001, *N*=3 differentiations). The majority of healthy astrocytes (Figure 2g; 61.2%±6.64; 93/145 cells) displayed long (defined as >1 × cell body width) branching fine processes consistent with archetypal astrocyte phenotype (herein referred to as ‘arborised' Figure 2e). A significantly smaller population showed a process-devoid, fibroblast-like phenotype (‘fibroblast-like' 24.9%±8.45; 33/145 cells; Dunn–Bonferroni *versus* arborised cells, *P*=0.002; Figure 2f, arrow). The remaining cells were thin, highly polarised, but essentially process-devoid cells (‘polarised' 13.9%±3.59; 19/145 cells; Dunn–Bonferroni *versus* arborised cells, *P*=0.017; *versus* process-devoid cells, NS; Figure 2f, arrowhead). In contrast, FAD and SAD astrocytes displayed reduced morphological heterogeneity compared with healthy cells. Specifically, FAD astrocytes comprise a significantly greater proportion of fibroblast-like cells than any other morphological type (Figure 3a, quantified in Figure 3e; fibroblast-like cells, 97.0%±1.38; 150/156 cells; Kruskal–Wallis, H_(2)_=10.091, *P*=0.006; Dunn–Bonferroni *versus* arborised cells, *P*=0.047; *versus* polarised cells, *P*=0.008; *N*=3 differentiations), despite their expression of mature astrocyte markers. This proportion of fibroblast-like cells was significantly greater than that seen with healthy astrocytes (Figure 5a; H_(2)_=14.530, *P*=0.001; Dunn–Bonferroni, *P*=0.008). FAD astrocytes with an archetypal astrocyte-like arborised morphology were rare and their relative proportion was significantly reduced in comparison with healthy control cells (1.94%±0.84; 4/156 cells; Kruskal–Wallis, H_(2)_=14.839, *P*=0.001; Dunn–Bonferroni, *P*=0.011). Similarly, SAD astrocyte cultures comprise mainly process-devoid fibroblast-like cells (Figure 4a; quantified in Figure 4e; 96.4%±2.64; 126/130 cells) with significantly fewer arborised cells (2.72%±2.72; 3/130 cells; Kruskal–Wallis, H_(2)_=11.129, *P*=0.004; Dunn–Bonferroni *versus* fibroblast-like, *P*=0.013; *N*=3 differentiations) or polarised cells (0.87%±0.87; 1/130 cells; Dunn–Bonferroni *versus* fibroblast-like, *P*=0.010). Again, there was a significant increase in the proportion of fibroblast-like cells in SAD astrocytes compared with healthy astrocytes (Figure 4a; Dunn–Bonferroni, *P*=0.004) concomitant with a reduction in the proportion of arborised cells (Dunn–Bonferroni, *P*=0.002). Notably, there was no significant difference in the relative proportions of each morphological cell type between the FAD and SAD astrocyte groups. Moreover, no significant difference in immunoreactive GFAP intensity was seen in any group (Kruskal–Wallis, H_(2)_=4.496, *P*=NS; Figure 5f), nor was any loss of nuclear integrity or fragmentation of the GFAP cytoskeleton observed. Taken together, these data demonstrate that astrocytes derived from patients carrying a *PSEN1* M146L mutation or both ApoE4 alleles display a significant reduction in morphological heterogeneity when compared with those derived from healthy individuals.

### Induced astrocytes from FAD and SAD patients display cellular atrophy

**Figure 6:**
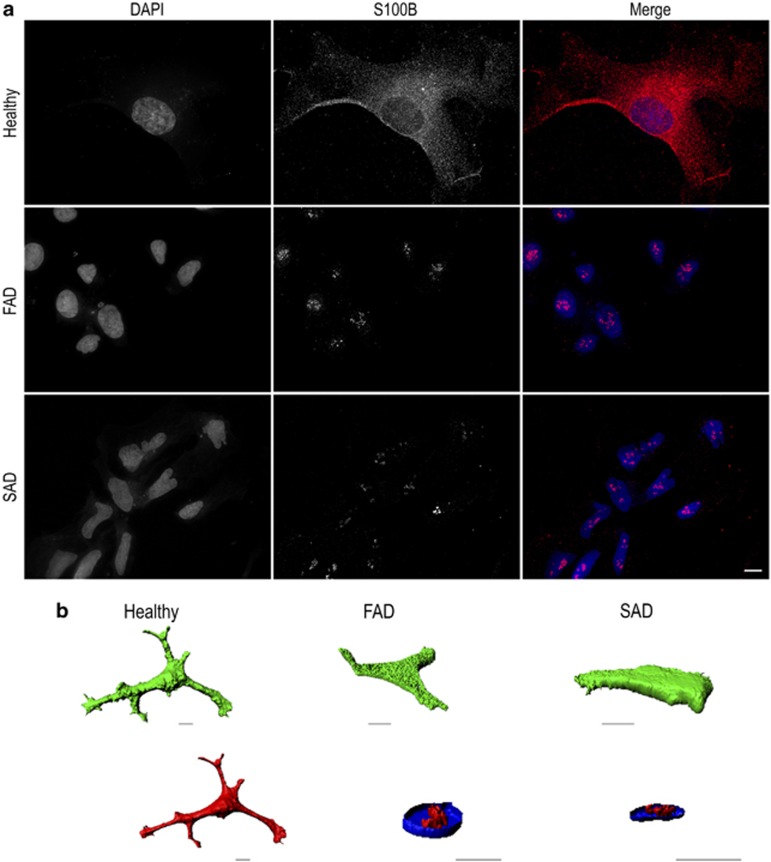
S100B localisation within astrocytes is dramatically altered in FAD and SAD Healthy astrocytes were immunostained for S100B and visualised using deconvolution fluorescence microscopy (**a**) revealing S100B to be distributed throughout the cytoplasm (top panel). In contrast, in FAD and SAD cells, S100B appeared to be localised exclusively at the nucleus (middle and bottom panels, respectively). Scale bar, 20 *μ*m. IsoSurface renders of S100B (red) and of DAPI-stained nuclei (blue; with a clipping plane set to permit visualisation of the inside of the nucleus) were constructed from serial confocal z-sections to further investigate the subcellular localisation of S100B (**b**; scale bars, 10 *μ*m). GFAP staining (green) is shown for comparison for healthy cells to reveal the overall cell morphology. In healthy cells, as expected, S100B displayed a cytosolic distribution, similar to that of GFAP. In both FAD and SAD astrocytes, S100B is localised exclusively to discrete foci within the nucleus. Nuclei images have been zoomed in × 2 for clarity. Movies of these renders can be viewed in Supplementary Movies S1–S3

Three-dimensional reconstruction of the entire GFAP cytoskeleton of astrocytes (Figure 6b top and Supplementary Movie S1) permitted comparisons of surface area and volume (Figures 5c and d). We found significant differences between healthy, FAD and SAD astrocytes in both their surface area and volume (surface area, Kruskal–Wallis; H_(2)_=48.454, *P*<0.001; volume, Kruskal–Wallis; H_(2)_=48.085, *P*<0.001). Specifically, FAD astrocytes displayed a significantly reduced GFAP surface area and volume (2220.4±204.6 *μ*m^2^ and 2574.4±307.5 *μ*m^3^, respectively; *N*=3 per group) in comparison with healthy astrocytes (9978.0±1048.0 *μ*m^2^ and 20867.7±2102.2 *μ*m^3^, respectively; *N*=3; Dunn–Bonferroni pairwise comparisons: surface area, *P*<0.001; volume, *P*<0.001). There was also a significant reduction in GFAP surface area and volume in SAD astrocytes (1853.5±171.0 *μ*m^2^ and 2301.7±270.2 *μ*m^3^, respectively; *N*=3 per group) when compared with healthy astrocytes (Dunn–Bonferroni pairwise comparisons: surface area, *P*<0.001; volume, *P*<0.001). No significant difference was observed in either the surface area or volume between FAD and SAD astrocytes (Figures 4c and d), suggesting that astrocytic atrophy may be a common feature in both forms of the disease.

To quantify astrocyte arborisation, we calculated the surface area to volume ratio, SA:Vol (Figure 5e). A larger SA:Vol ratio would indicate a cell with a relatively small cell body (low volume) displaying many processes (large surface area), whereas a small SA:Vol ratio would indicate a more rounded, fibroblast-like cell (large volume) lacking processes (low surface area). We found that the SA:Vol ratio was significantly higher in healthy astrocytes (2.12±0.07 *μ*m^−1^) compared with either FAD or SAD cells (1.14±0.05 *μ*m^−1^ and 1.19±0.05 *μ*m^−1^, respectively; Kruskal–Wallis; H_(2)_=47.385,*P*<0.001, Dunn–Bonferroni pairwise comparisons: healthy *versus* FAD and SAD, both *P*<0.001; FAD *versus* SAD, *P*=NS), indicating a greater degree of arborisation in healthy over diseased cells. Again, these data reveal very little difference between FAD and SAD astrocytes, indicating that aberrant astrocyte morphology is a shared feature of both forms of the disease.

### Aberrant expression and localisation of S100B, EAAT1 and GS in AD astrocytes

In healthy astrocytes, S100B expression was observed throughout the cytoplasm and, to a lesser extent, in the nucleus, consistent with normal astrocytes *in vivo* (Figure 6a, top panel; Figure 6b, left; Supplementary Movie S1). In contrast, both FAD and SAD astrocytes displayed multiple large accumulations of S100B localised to the nucleus, with very little staining in other cellular compartments (Figure 6a, middle and bottom panels, respectively). To confirm that S100B was confined within the nuclei of diseased cells, we utilised z-sectioning confocal microscopy and IsoSurface reconstruction to render 3D models of the staining. Renders of GFAP and DAPI staining were also made to permit comparisons. In both FAD and SAD astrocytes, S100B expression was restricted to the nucleus and confined to multiple, discrete foci following a similar pattern to that of nucleoli (Figure 6b, centre and right; Supplementary Movies S2 and S3, respectively). Fluorescence intensity analysis indicated that S100B levels were significantly reduced in both FAD and SAD compared with controls, but unchanged relative to each other (ANOVA, F_(2,16)_=44.509, *P*<0.001; Hochberg's GT2, healthy *versus* FAD and SAD *P*<0.001, FAD *versus* SAD *P*=NS; Figure 5g). In both healthy and FAD astrocytes, EAAT1 revealed a uniformed pattern of fluorescence extended throughout the cell (Figures 2c and 3c, respectively). In contrast, SAD astrocytes exhibited confined nuclear accumulation of EAAT1 (Figure 4c). Quantification of fluorescence intensity revealed that EAAT1 levels were lower in both FAD and SAD astrocytes compared with healthy cells, although only FAD differed significantly (Kruskal–Wallis; H_(2)_=4.235, *P*=0.001; Bonferroni, healthy *versus* FAD, *P*<0.001, *versus* SAD, *P*=NS; Figure 5h). GS immunoreactivity in healthy astrocytes revealed a uniform pattern of fine puncta throughout the cytoplasm (Figure 2d, inset). In diseased cells, however, this smooth pattern was lost and replaced with a distinctly more clumped pattern of staining with much larger puncta (FAD, Figure 3d, inset; SAD, Figure 4d, inset). Fluorescence intensity analysis revealed GS levels were lower in both FAD and SAD astrocytes compared with healthy cells, although only FAD differed significantly (Kruskal–Wallis; H_(2)_=13.430, *P*=0.001; Bonferroni, healthy *versus* FAD, *P*=0.001, *versus* SAD, *P*=NS; Figure 5i). Overall these data reveal a drastic reorganisation of key proteins associated with astrocyte function in both FAD and SAD.

### Altered non-stimulated release of soluble inflammatory mediators in AD Astrocytes

Non-stimulated astrocytes were cultured under adherent culture conditions and astrocyte conditioned medium (ACM) simultaneously probed for 36 soluble mediators (Supplementary Table S1) using a proteomic array (Supplementary Figures S2A–C). Astrocytes, irrespective of their genotype, constitutively secreted only three cytokines: IL-8 (CXCL8), MCP-1 (CCL2) and TIMP-2. However, SAD astrocytes additionally secreted RANTES (CCL5) and MIP-1β. Quantification of expression levels using densitometry showed that IL-8 and MCP-1 secretion was significantly decreased in both non-stimulated ACM from FAD- and SAD-derived astrocytes compared with control ACM (Dunn–Bonferroni; *P*<0.001 for both, *N*=3; Supplementary Figure S2D). These results show that human astrocytes secrete a distinct set of soluble mediators under non-stimulated physiological conditions *in vitro* and are partially compromised in both FAD and SAD astrocytes.

## Discussion

**Figure 7:**
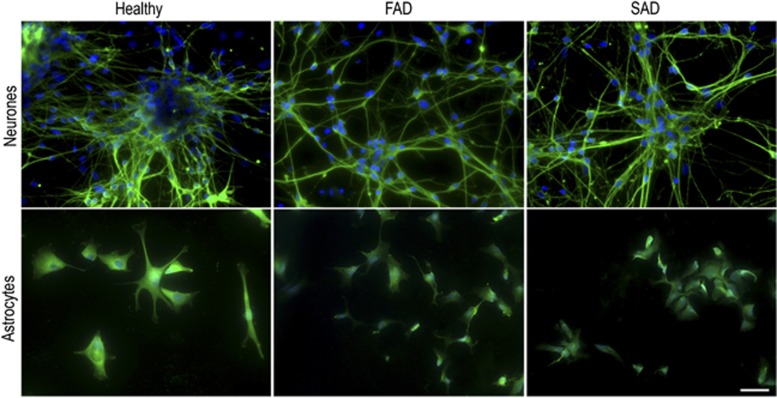
Comparison of healthy control, FAD and SAD patient-derived *β*III-tubulin immunoreactive neurones and GFAP immunoreactive astrocytes. Early neuronal appearance is indistinguishable across the groups, whereas AD astrocytes show markedly reduced heterogeneity of morphology and striking atrophy compared with healthy cells. Scale bar, 50 *μ*m

Since astrocytes are the main homeostatic units of the CNS, their dysfunction may drive progression of neurologic disorders, including AD. Astrocytes contribute to non-cell autonomous mechanisms in various neurodegenerative disorders that were previously thought of as classically neuronal diseases.^28, 29^ Here we report novel pathological astroglial phenotypes in both FAD and SAD through exploitation of an iPSC-based human model. We show evidence to support critical astroglial contribution to AD namely: (i) mislocalisation and abnormal expression of mature astrocyte markers, (ii) compromised astrocyte heterogeneity and (iii) astroglial atrophy. Singularly, we did not observe differences in early maturation of AD-derived neurones compared to healthy controls, showing that astroglial changes are cell autonomous and do not derive from compromised neuronal intermediates (Figure 7). Generation of functional astrocytes from healthy iPSCs has previously been reported to be time-consuming and result in 20–30% contamination of unwanted cell types.^18, 19, 20^ In contrast, we demonstrate highly efficient generation of enriched populations of mature cortical astrocytes (>95%), irrespective of donor origin, within 30 days of induction. This is likely due to additional medium supplementation of EGF and insulin, both of which have been previously shown to increase glial commitment and primary astrocyte proliferation *in vitro*.^26, 27^ Owing to a lack of astrocyte markers that allow precise examination of subtypes that occur throughout the CNS, we were not able to confirm their cortical identity. However, it has been previously published that maturation of neurones or glia from regionally specified iPSC-derived NPCs maintain their identity.^30^ Critically, the healthy iPSC-derived astrocytes described herein were comparable in their morphology and marker expression to those described in several previously published independent studies; validating our differentiation protocol and model.^18, 19^

Astrocytes exhibited 95–98% expression of GFAP, EAAT1, GS and S100B irrespective of healthy or disease origin. However, we observed significant redistribution and decreased expression of S100B in FAD and SAD astrocytes compared with healthy cultures. Although nuclear S100B expression has previously been reported,^18, 19, 20^ its expression has not been shown to be exclusively restricted to the nucleus/nucleoli. Why S100B would localise to a nuclear sub-compartment in pathologically remodelled astrocytes remains to be established, but given that S100B is known to interact with various cytoskeletal components, together with the reduction in its expression levels, may represent a novel and early mechanism underlying SAD- and FAD-induced astrocytic atrophy. Mislocalisation of astrocytic GS was also observed in FAD and SAD astrocytes, however, altered subcellular EAAT1 distribution was only observed in cultures derived from SAD cells. Previous studies have shown altered glutamatergic signalling in neuronal AD degeneration.^31, 32, 33^ Our results further reveal a previously unrecognised astroglial cell autonomous pathological phenotype in AD, although a significant reduction in the overall fluorescent intensities of EAAT1 and GS was only seen in FAD-derived astrocytes. This finding echoes our recent report of a significant decrease in GS expression in the frontal cortex of 3xTg-AD murine model,^34^ and may hint that astroglia-associated glutamate turnover differs between the two forms of AD at an early stage.

We found that healthy induced astrocytes show significant morphological heterogeneity, in accordance with morphologically distinct cortical astrocytic subpopulations *in vivo*,^35^ further validating the use of our cell-based model for the interrogation of astroglial function. In contrast, FAD and SAD astrocytes have reduced heterogeneity, are significantly smaller than their healthy counterparts and exhibit an almost complete absence of processes. This corroborates recent studies in mouse models of FAD showing reduced somata and process volumes in astrocytes together with a reduction in the number and complexity of astrocyte processes are an early feature of the disease.^10, 11, 36, 37^ Our results confirm that cell autonomous astrocytic atrophy is a feature of early-onset FAD, but also provide evidence that astrocytic aberrance is a likely characteristic of SAD.

We also show evidence for an altered basal astrocyte secretome profile in both FAD- and SAD- derived cells. Our study of non-stimulated healthy astrocytes in culture express a similar array of inflammatory mediators previously identified in cultures from highly purified human fetal and adult astrocytes.^38, 39^ Furthermore, these mediators have been previously shown to be direct targets of the transcription factor NF-*κ*B indicating that this pathway may be constitutively active in human astrocytes under two-dimensional culture conditions.^38, 39^ We also observed significantly compromised constitutive secretion of the pro-inflammatory mediators; IL-8 (CXCL8) and MCP-1 (CCL2) in FAD- and SAD-derived ACM, both of which have been previously shown to be the most abundantly expressed transcripts in adult healthy human astrocytes *in vitro*.^39^ These early observations may indicate a potential glial paralysis as such, which has been postulated to be a fundamental factor in the evolution of AD.^14^ Although, herein we describe the use of a single patient-derived cell line for each criteria, it is reasonably common-place that articles in this field are published whereby studies describe only a single patient-derived cell line due to the labour-intensive and cost-prohibitive nature of induced pluripotent stem cell-derived culture methods and differentiation protocols.^17, 40, 41, 42, 43, 44, 45^

However, it should be emphasised that cultured astrocytes utilised in the present study represent a simplified model relative to that of astrocytes in the CNS, whereby additional interactions with other cell types and matrix components are likely to influence the astrocytic phenotype. Yet, our highly purified human astrocyte culture model presents a unique system to delineate the autonomic responses of astrocytes to defined stimuli/matrix/co-cultures in both healthy and AD-affected cells.

## Conclusions

Using a human *in vitro* iPSC model of familial and sporadic AD, we demonstrate a significant and previously unknown cell autonomous pathological phenotype of astroglia. Crucially, neuronal intermediates derived from identical iPSCs obtained from the same patients, did not show any pathological phenotype when compared with healthy controls, further corroborating recent ideas of the fundamental role of astroglia in the development of neurodegenerative diseases. We may further conjecture that early synaptic dysfunction, arising from the inhibition of astrocyte–synapse interplay and disruption of functional astroglial networks, is an early feature of AD progression, supporting the idea of astrocytic atrophy as a plausible mechanism for early cognitive impairment and thus providing a potential novel therapeutic target for AD intervention.

## Materials and methods

All reagents were purchased from ThermoFisher (Paisley, UK) unless stated otherwise.

### Derivation and maintenance of neuronal cell lineages

Human iPSC-derived cortical neural progenitor cells (NPCs) were supplied by Axol Bioscience (Cambridge, UK) and generated from patient dermal fibroblasts exhibiting an M146L mutation in the presenellin-1 gene (*PSEN1*) from a 53-year-old male donor clinically affected with type III early-onset FAD and from an 87-year-old female clinically affected with late-onset SAD homozygous for the four allele of apolipoprotein E (*ApoE4*^+/+^). The control samples were supplied by Axol Bioscience and reprogrammed from human female umbilical cord blood cells using the same method. NPCs were expanded following manufacturer guidelines and expression of known NPC markers were confirmed before astroglial differentiation.

For directed astroglial differentiation, NPCs were dissociated using accutase and attached with a substrate of poly-l-ornithine (20 *μ*g/ml; Sigma, Dorset, UK) and mouse laminin (10 *μ*g/ml) in chemically defined medium modified from Shaltouki *et al.*^18^ comprising neurobasal medium containing B27 supplement (1 ×), non-essential amino acids (1 ×), l-glutamine (2 mM), penicillin/streptomycin (50 U/ml), 8 ng/ml FGF2 (Peprotech, London, UK), 5 ng/ml CNTF and 10 mg/ml BMP2 (Peprotech) with additional supplementation of 10 ng/ml EGF and 5 *μ*g/ml insulin. NPCs were seeded at 3 × 10^4^ cells/cm^2^ and maintained at 37 °C/5% CO_2_. The cells were cultured until 80% confluence (approximately 5 days) and passaged twice, as previously detailed. On the third passage, and all consecutive passages, the cells were plated onto non-coated tissue culture plastic to remove unwanted neurones and enrich for astroglia due to their superior adhesiveness. The cells were passaged at least three times during astroglial commitment. Astrocytes were generated a minimum of three times using independent differentiations from each patient sample.

For terminal neuronal differentiation, NPCs were seeded as above, but onto plates coated with 20 *μ*g/ml poly-l-ornithine and 10 *μ*g/ml laminin. The cells were maintained in a 1:1 mixture of N2:B27 media; where N2 medium comprised; DMEM/F-12 with Glutamax, N2 supplement (1 ×), 1 mM l-glutamine, non-essential amino acids (1 ×), 0.1 mM *β*-mercaptoethanol, 50 U/ml penicillin/streptomycin and 5 *μ*g/ml insulin (Sigma); and B27 medium comprised Neurobasal, B27 supplement (1 ×), 200 mM l-glutamine, 50 U/ml penicillin/streptomycin. Terminally differentiated neurones were maintained for 30–40 days with medium changes every other day. The cell counts and viability were performed using Trypan blue exclusion assay and calculated using an automated cell counter. Population doubling times were calculated when viable cells exhibited exponential growth. Neurones were generated a minimum of three times using independent differentiations from each patient sample.

### Immunocytochemistry

The cells were fixed in 4% (w/v) paraformaldehyde in phosphate-buffered saline (PBS) for 15 min and then treated with 0.1 M glycine in PBS for a further 10 min to quench unreacted aldehydes. Fixed cells were permeabilised by incubation in 0.1% (w/v) Triton X-100 in PBS for 5 min and blocking of nonspecific binding was achieved by incubation with 1% (w/v) bovine serum albumin (BSA) in PBS for 1 h. The cells were subsequently incubated for 30 min in 1% BSA/PBS containing rabbit polyclonal IgG antiserum for the glial fibrillary acidic protein (GFAP; 1:100; Sigma), mouse monoclonal clone 1B2 IgG1*κ* for S100 calcium-binding protein B (S100B; 1:100; Sigma), rabbit polyclonal IgG antiserum for the excitatory amino acid transporter-1, (EAAT1; 1:200; Abcam, Cambridge, UK), goat polyclonal antiserum for glutamine synthetase (GS; C-20; 1:100; Santa Cruz Biotechnology, Dallas, TX, USA), rabbit polyclonal IgG antiserum paired box-6 (PAX6; 1:100; BioLegend, London, UK), recombinant monoclonal rabbit IgG sex determining region-1 (SOX1; 1:100; Abcam), monoclonal mouse IgG1 nestin (1:200; Abcam) or COUP-TF-Interacting Protein 2 (CTIP2; 1:300; Abcam). Appropriate Alexa 488 or Alexa 546-conjugated fluorescent secondary antibodies in 1% BSA/PBS with 0.1 *μ*g/ml DAPI were applied (Sigma) for a further 30 min. Coverslips were mounted with ProLong Diamond antifade mountant, cured overnight and sealed with nail varnish before imaging.

### Fluorescence microscopy and image analysis

The fixed cells were visualised using a Zeiss Cell Observer z-sectioning fluorescence imaging system equipped Zeiss definite focus, HXP 120 V illumination and FITC, DsRed and DAPI filter sets. Fluorescence images were acquired using × 20 PL Apo (0.8  NA), × 40 EC Plan-Neofluar (1.3 NA) oil and × 63 PL Apo (1.4 NA) oil objectives, AxioCam MRm Rev.3 CCD camera and ZEN Pro software (Carl Zeiss, Cambridge, UK). The images were collected as serial optical z-sections taken at 0.28 *μ*m intervals, which were subsequently deconvolved using a fast iterative algorithm with a maximum of five iterations. The z-stacks were processed and analysed using FIJI ImageJ.^46, 47^ All the images presented herein are maximum intensity projections of these z-stacks unless otherwise indicated. Morphological analysis was carried out by visually binning cells into one of three categories: fibroblast-like process-devoid cells, process bearing arborised cells and polarised cells. A minimum of 130 cells drawn from three separate batches of differentiated cells were analysed at × 20 magnification. Immunofluorescence intensity analysis was performed on a minimum of five random fields for each of three separate batches of differentiated cells of view at × 20 magnification using Fiji ImageJ. Z-stacks were flattened before the average background fluorescence, calculated from three random regions of each field of view, was subtracted. Raw integrated pixel densities of each image were calculated and divided by the number of immunoreactive cells to determine an average integrated pixel density per cell for each group.

To permit quantification of GFAP cytoskeleton volume and surface area, serial optical z-sections were collected at 0.5 *μ*m intervals using a Leica TCS SP5 AOBS confocal microscope equipped with a × 63 HCX PL Apo (0.60–1.40 NA) oil objective, 405 nm and 488 nm laser lines and internal PMTs (Leica Microsystems (UK) Ltd, Milton Keynes, UK). Throughout all experiments, the pinhole was set to one Airy unit. The images were collected sequentially to minimise crosstalk using a bidirectional scan at 1000 Hz and three frame averages. Z-stacks were carefully created so as to include the entire thickness of each cell. Images were subsequently analysed using Imaris 7.7.2 3D visualisation software (Bitplane AG, Zurich, Switzerland) using the IsoSurface tool to create a smoothed 3D surface render of each cell by demarcating the edges of the GFAP-positive cytoskeleton and removing voxels before calculating GFAP surface area, volume and mean fluorescence intensity. A similar approach was used to determine the subcellular localisation of S100B by creating 3D surface renders of both S100B staining and DAPI-stained nuclei, before using a clipping plane to slice the DAPI channel only, revealing S100B staining within the nucleus. In each experimental group, a minimum of 21 randomly selected cells drawn from three separate differentiations were analysed. To quantify neural progenitor-, neurone- and astroglial-marker expression, the cell counts were expressed as a percentage of total cells in a field of view, where the total number of cells was identified using DAPI-stained nuclei. The images were captured under standard epifluorescence on an Olympus IX71 microscope (Olympus, Southend-on-Sea, UK) and analysed using ImageJ. Five randomly chosen fields from three independent experiments were assessed.

### Antibody arrays

ACM was collected following 30 days in culture and stored at −80 °C analysis of inflammatory mediators using a human antibody array kit (Abcam). ACM was collected following cell exposure for 48 h (normalised to cell number) and samples analysed in parallel to enable comparisons in relative expression levels between experiments. Each experiment represents pooled medium from three independent differentiations. Before analysis, all the samples were thawed on ice and centrifuged at 10 000 × *g* for 5 min at 4 °C. Cytokine/chemokine secretion was measured according to the manufacturer's instructions. Since, recombinant growth factors are present in ACM, a media-only control was also run in parallel to normalise the data. Positive immunoreactivity was visualised using chemiluminescence using a CCD camera (Bio-Rad, Hertfordshire, UK) and positive/negative membrane controls permitted densitometric analysis using Imagelab software (Bio-Rad).

### Statistical analysis

All statistical analyses were carried out using IBM SPSS 22 (IBM Corp., Armonk, NY, USA). Data distributions were analysed by plotting histograms and Quantile–Quantile plots followed by Shapiro–Wilks tests to determine normality. For cell derivation and population analyses, each experiment was performed a minimum of three times and all assays were repeated in triplicate. Mean proportions of nestin+ NPCs and *β*III-tubulin+ neurones were compared by one-way ANOVA. Analyses of cell viability and proliferation rates, cell morphologies and immunofluorescence intensity (with the exception of S100B) was carried out using Kruskal–Wallis tests followed by pairwise comparisons by the Dunn–Bonferroni method. S100B immunofluorescence intensity comparison was carried out by ANVOA followed by a Hochberg's GT2 *post hoc*. All the data are presented as mean±standard error. An alpha level of 0.05 was considered significant throughout.
