# Correction: Aberrant iPSC-derived human astrocytes in Alzheimer’s disease

**DOI:** 10.1038/s41419-019-1422-7

**Published:** 2019-03-12

**Authors:** V. C. Jones, R. Atkinson-Dell, A. Verkhratsky, L. Mohamet

**Affiliations:** 1 The University of Central Lancashire, Preston, PR1 2HE UK; 2 The University of Manchester, Manchester, M13 9PT UK; 3 Achucarro Center for Neuroscience, IKERBASQUE, Basque Foundation for Science, Bilbao, 48011 Spain

**Correction to:** **Cell Death & Disease** 8:e2696

10.1038/cddis.2017.89

published online 23 March 2017

The original version of this Article contained an error in Fig. 1, in which a number of incorrect fluorescence images were inadvertently incorporated into the panel. The conclusions of the paper are not changed in any way by this correction, and the authors apologize for the inconvenience.

The correct version of Fig. 1 is:

**Fig. 1:**
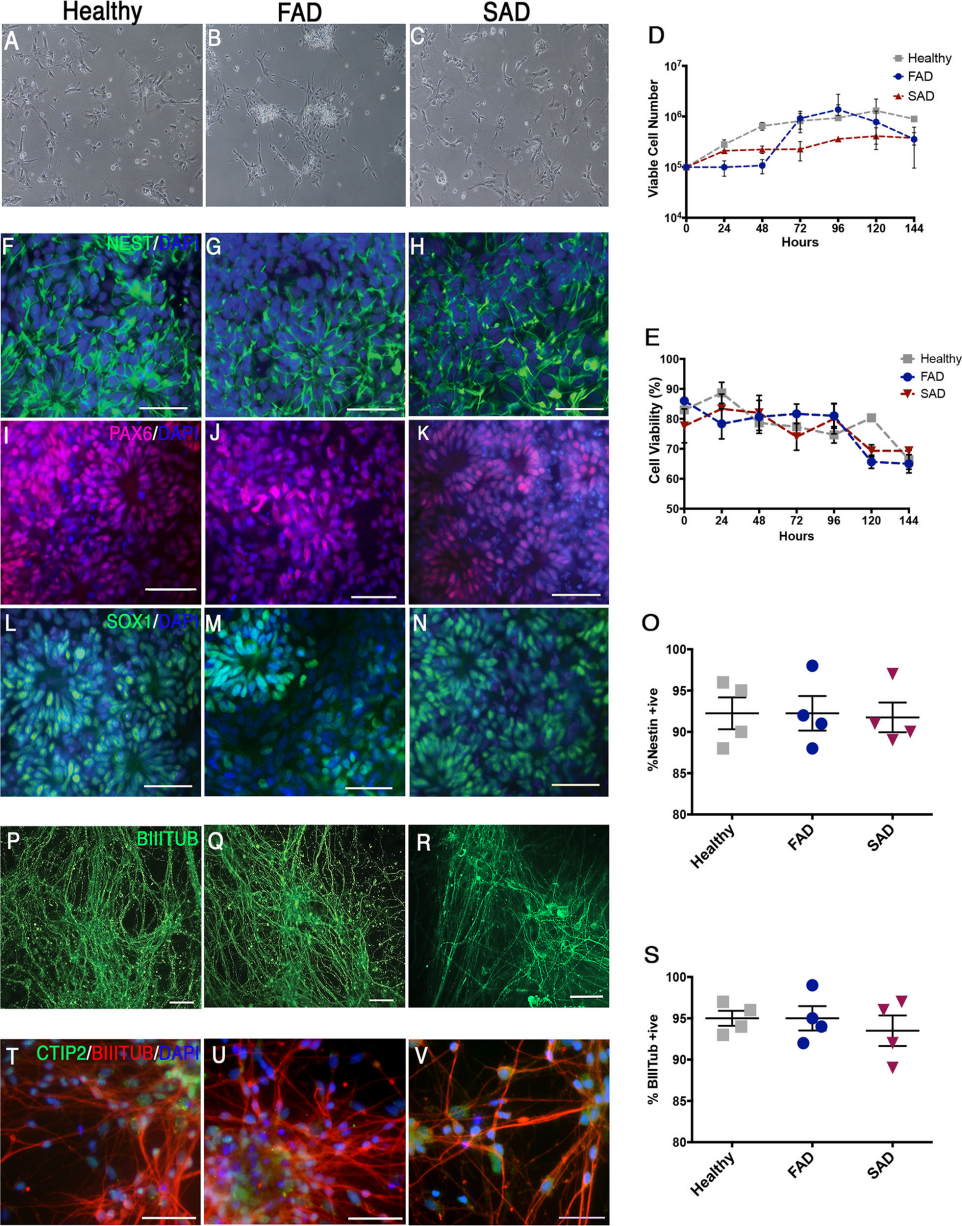
Directed differentiation of healthy and AD-neural progenitor cells into cortical neurones. **a**–**c** NPCs were seeded at 1 × 10^5^ per well and propagated in monolayer culture for 6/7 days. FAD and SAD cortical NPCs showed indistinguishable culture morphology with healthy (control) NPCs (*N* = 5 per cell line). **d** and **e** No significant differences in NPC growth rates were identified (*N* = 4 per cell line, two-way Kruskal–Wallis *P* = NS). **f**–**n** IPS-derived NPCs from healthy (control), FAD and SAD patients were assessed for canonical marker expression. Progenitor cells formed polarised rosettes expressing nestin (green; **f**–**h**), PAX6 (red; **i**–**k**) and SOX1 (green, **l**–**n**). **o** No significant difference in nestin+ cells was observed between healthy and AD cell lines (*N* = 4 per cell line, ANOVA, *F*_(2,9)_ = 0.022, *P* = NS). **p**–**r** Under terminal neuronal differentiation conditions for 35–40 days, all patient samples showed positive expression of the neural marker *β*-III-tubulin (green). **s** No significant difference in the proportion of *β*-III-tubulin+ neurones between any individual (*N* = 4 per group, ANOVA, *F*_(2,9)_ = 0.128, *P* = NS). **t**–**v** Expression of the mature deep-layer cortical neuronal marker, CTIP2 was observed throughout cultures from each patient (green). Scale bars, 50 μm

This has been corrected in both the PDF and HTML versions of the Article.

