# Supplementary figures and images for: Aberrant iPSC-derived human astrocytes in Alzheimer's disease
Source: Cell Death Dis. 2017 Mar 23;8(3):e2696–. doi: 10.1038/cddis.2017.89 (PMC5386580; doi:10.1038/cddis.2017.89)

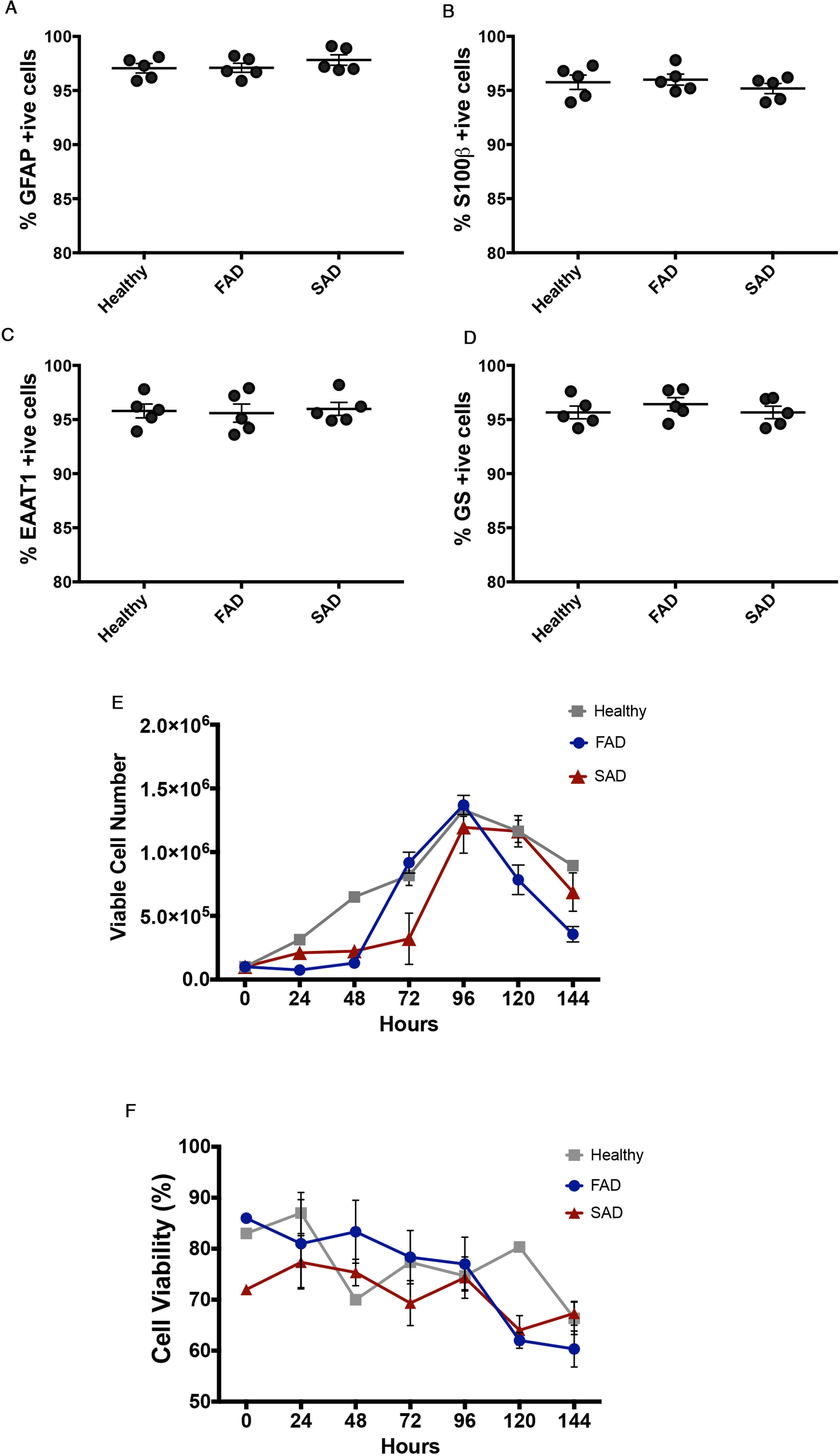

Supplement: Supplementary Figure S1 [file cddis201789x1.tif]

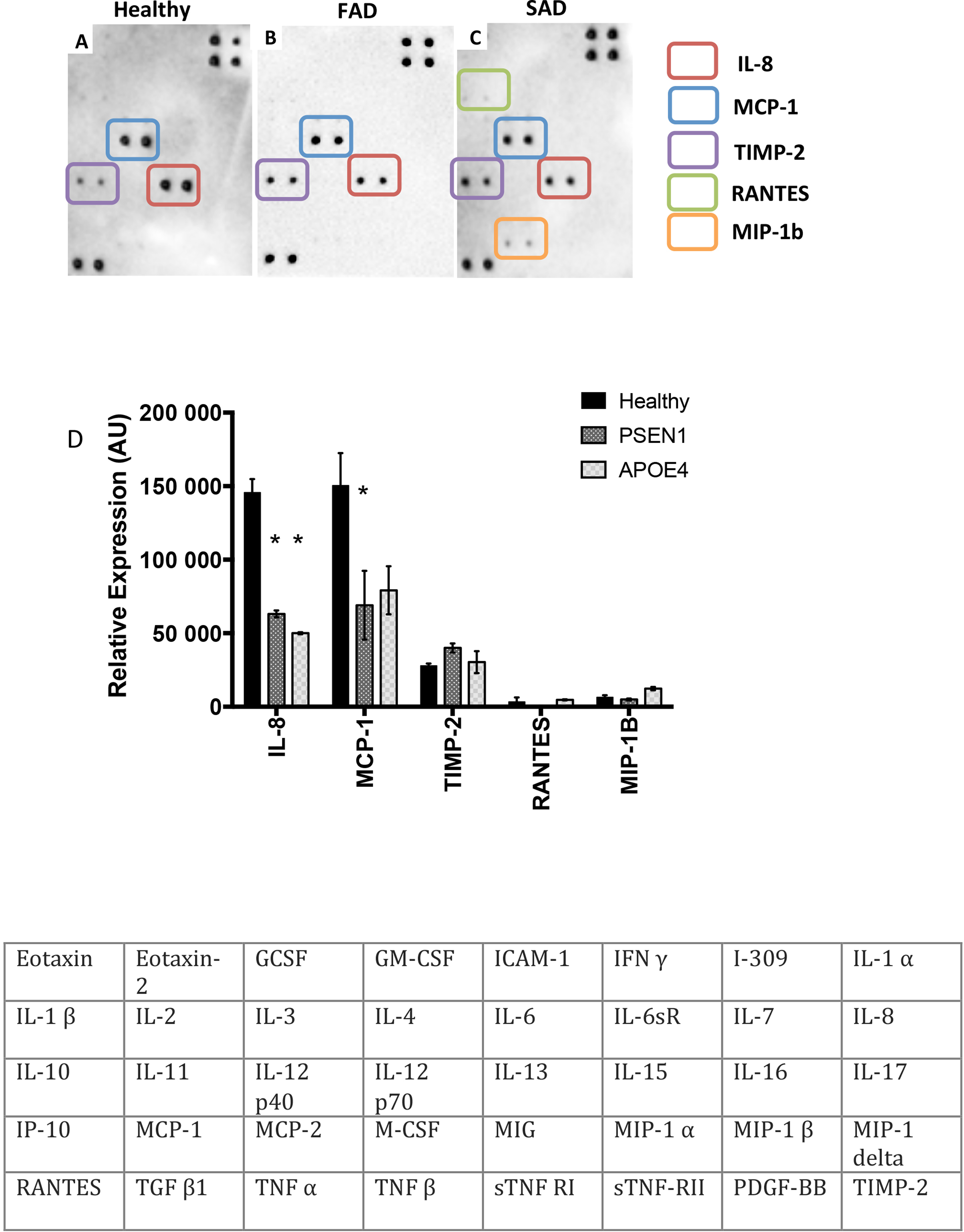

Supplement: Supplementary Figure S2 [file cddis201789x2.tif]
